# Chronic Inflammation in Ulcerative Colitis Causes Long-Term Changes in Goblet Cell Function

**DOI:** 10.1016/j.jcmgh.2021.08.010

**Published:** 2021-08-18

**Authors:** Varsha Singh, Kelli Johnson, Jianyi Yin, Sun Lee, Ruxian Lin, Huimin Yu, Julie In, Jennifer Foulke-Abel, Nicholas C. Zachos, Mark Donowitz, Yan Rong

**Affiliations:** 1 Division of Gastroenterology & Hepatology, Department of Medicine, Baltimore, Maryland; 2 Department of Cellular and Molecular Physiology, Johns Hopkins University School of Medicine, Baltimore, Maryland

**Keywords:** Ulcerative Colitis, Goblet Cell, Mucus Layer, Colonoids, ATOH1, atonal homolog 1, cAMP, cyclic adenosine monophosphate, Cch, carbachol, DF, differentiated, 3-D, three-dimensional, GC, goblet cell, HS, healthy subjects, IBD, inflammatory bowel disease, NDM, non-differentiated medium, *Ngn 3*, Neurogenin 3, PCR, polymerase chain reaction, PGE_2_, prostaglandin E_2_, SEM, standard error of the mean, *SPEDF*, SAM pointed domain containing Ets transcription factor, TEER, transepithelial electrical resistance, TEM, transmission electron microscopy, TNF, tumor necrosis factor, UC, ulcerative colitis, UD, undifferentiated

## Abstract

**Background & Aims:**

One of the features of ulcerative colitis (UC) is a defect in the protective mucus layer. This has been attributed to a reduced number of goblet cells (GCs). However, it is not known whether abnormal GC mucus secretion also contributes to the reduced mucus layer. Our aims were to investigate whether GC secretion was abnormal in UC and exists as a long-term effect of chronic inflammation.

**Methods:**

Colonoids were established from intestinal stem cells of healthy subjects (HS) and patients with UC. Colonoids were maintained as undifferentiated (UD) or induced to differentiate (DF) and studied as three-dimensional or monolayers on Transwell filters. Total RNA was extracted for quantitative real-time polymerase chain reaction analysis. Carbachol and prostaglandin E_2_ mediated mucin stimulation was examined by MUC2 IF/confocal microscopy and transmission electron microscopy.

**Results:**

Colonoids from UC patients can be propagated over many passages; however, they exhibit a reduced rate of growth and transepithelial electrical resistance compared with HS. Differentiated UC colonoid monolayers form a thin and non-continuous mucus layer. UC colonoids have increased expression of secretory lineage markers *ATOH1* and *SPDEF*, along with MUC2 positive GCs, but failed to secrete mucin in response to the cholinergic agonist carbachol and prostaglandin E_2_, which caused increased secretion in HS. Exposure to tumor necrosis factor α (5 days) reduced the number of GCs, with a greater percentage decrease in UC colonoids compared with HS.

**Conclusions:**

Chronic inflammation in UC causes long-term changes in GCs, leading to abnormal mucus secretion. This continued defect in GC mucus secretion may contribute to the recurrence in UC.

## Graphical abstract

**Figure:**
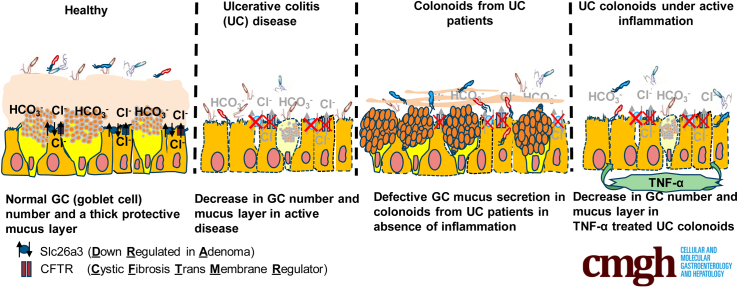

**Summary**

Our results suggest that the abnormal mucus layer in UC patients is due to the effect of an active inflammatory environment to reduce the number of goblet cell (GCs), as well as due to long-term changes in stimulated mucin secretion that persist even in the absence of inflammatory cells.

Ulcerative colitis (UC) is a chronic relapsing colonic disorder. A frequent colonic abnormality in UC is a reduced mucus layer secreted by goblet cells (GCs). Mucus layer defects contribute to the UC pathophysiology by triggering immune responses and/or allowing increased and more proximate exposure to luminal bacteria, both of which can lead to further reduced barrier maintenance, mucosal damage, defective absorption, and increased fluid secretion. The mucus layer is secreted by GCs, which primarily occur in differentiated (DF) colonocytes. Secretion of pro-inflammatory cytokines in UC contributes to the destruction of the epithelial barrier including the mucus layer.^1^, ^2^, ^3^, ^4^ However, even in the absence of endoscopic signs of active inflammation, the intestinal mucosa of UC patients in remission has a defective mucus layer and histologic changes including branching of crypts, thickened muscularis mucosa, Paneth cell metaplasia, and neuroendocrine cell hyperplasia.^5^ These changes suggest that the recovered intestine is permanently altered even after the inflammation has resolved. In fact, the intestinal epithelium of UC in remission has an expression profile that is significantly different from that found in healthy mucosa, which includes increases in expression of REG4, S100P, SERPINB5, DEFB1, and AQP3 and decreases in SLC16A1and AQP8 expression.^6^^,^^7^ Importantly, these genes modulate epithelial cell growth, sensitivity to apoptosis, and immune function.^7^ Other studies have shown that intestinal epithelium of inflammatory bowel disease (IBD) patients can harbor persistent alterations in gene expression or DNA methylation despite complete endoscopic and histologic remission.^2^^,^^8^^,^^9^ These changes could contribute to the frequent disease recurrences that are common in UC.^7^^,^^10^, ^11^, ^12^ Altogether these results support the view that changes in the mucosa of patients with UC persist long after the inflammation has resolved.

We hypothesized that a long-term consequence of colonic inflammation in UC is abnormal GC function that includes reduced stimulated mucus secretion. To test this hypothesis, we used an ex vivo human organoid/colonoid model made from healthy subjects (HS) and from active and inactive mucosa of UC patients. Our results suggest that UC colonoids, which lack the presence of inflammatory cells, maintain an abnormal GC phenotype, with a reduced mucus layer due to defective cholinergic/prostaglandin E_2_ (PGE_2_) induced mucus secretion but with an increase in the number of GCs. Exposure of UC colonoids to tumor necrosis factor (TNF)-α reduced the number of GCs, which occurred to a greater extent than in HS colonoids. Our results suggest that the abnormal mucus layer in UC is due to the effects of an active inflammatory environment to reduce the number of GCs as well as due to long-term changes in stimulated mucin secretion that persist even in the absence of inflammatory cells and exist in colonoids made from active and inactive UC.

## Results

### UC Derived Colonoids Can Be Grown in Culture Over Multiple Passages but They Exhibit a Reduced Growth Rate

**Figure 1:**
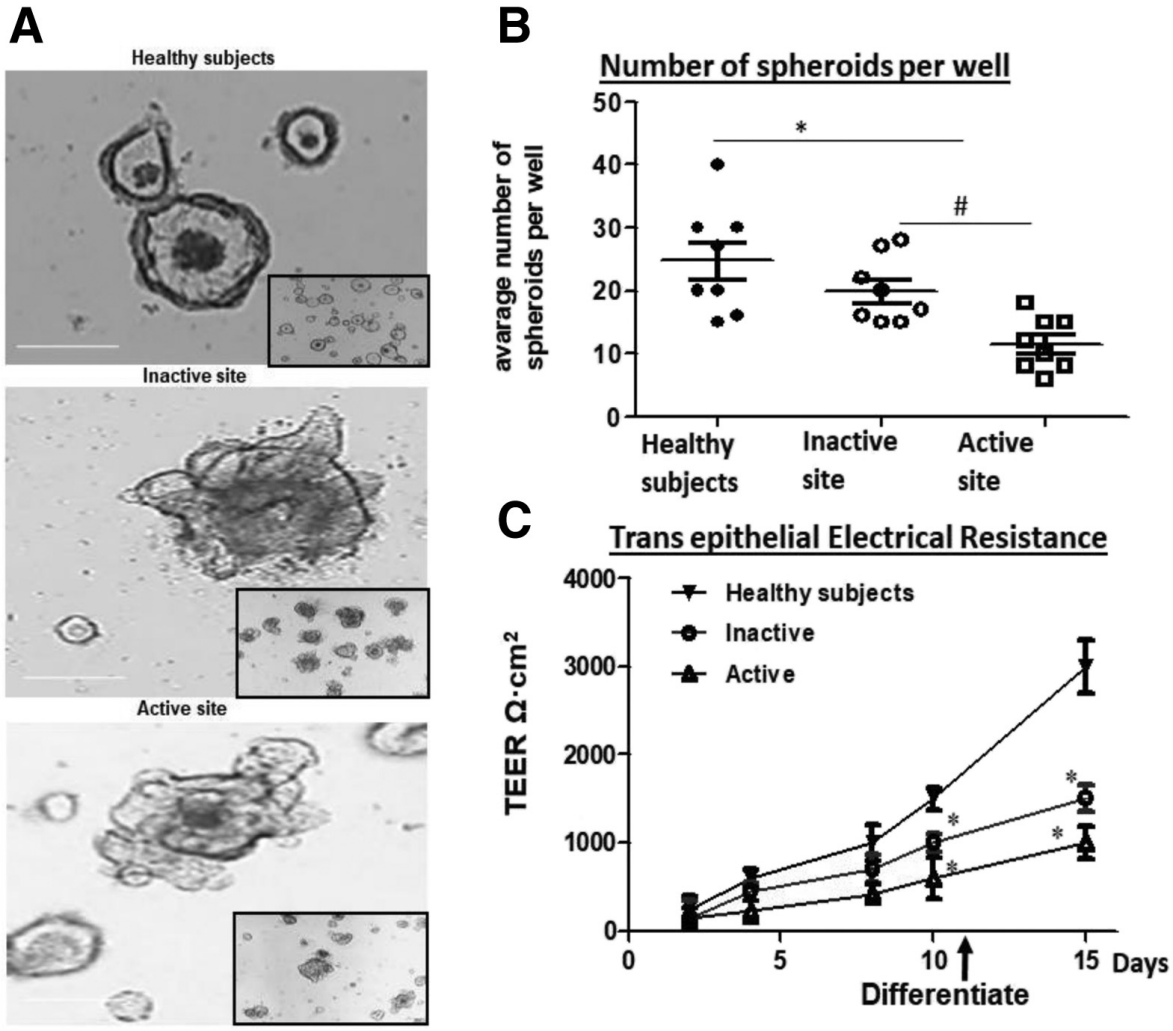
**UC colonoids have differences in growth compared with colonoids from HS.***(A)* A representative bright field image of 3D colonoids from HS and inactive and active sites of UC patients. *(B)* Number of 3D spheroids per well from HS and inactive and active sites of UC patients. Quantitation of spheroids was made 2 days after splitting. *(C)* Changes in TEER of colonoid monolayers from HS (*black triangle*), inactive UC (*circle*), and active (*triangle*) UC. Spheroids and monolayers from each subject were analyzed at least 3 times and used as n = 1. ∗*P* < .05 vs HS, ^#^*P* < .05 vs inactive UC. Scale bar = 20 μm.

Human UC patient-derived colonoids were propagated and compared with site-matched HS colonoids. Similar to colonoids grown from HS, UC colonoids formed three-dimensional (3D) spheroids and could be passaged at least 40 times. Figure 1*A* shows the phase-contrast images of colonoids from HS and UC patients 5 days after splitting. Morphologically, UC colonoids had more budding structures compared with HS. However, when the growth of 3D spheroids was quantitated by measuring the number of spheroids per well after each split over time and for multiple passages, active disease UC colonoids grew slowly and formed fewer spheroids compared with inactive UC and HS (Figure 1*B*). We have demonstrated that human colonoids can be grown as 2D monolayers.^13^ The progress of monolayer formation was monitored daily by a steady increase in transepithelial electrical resistance (TEER) (Figure 1*C*). The monolayers were maintained in the undifferentiated (UD) crypt-like state by growth in Wnt3A, RSPO1, and Noggin, whereas withdrawal of growth factors (Wnt3A and RSPO1) drove differentiation by 5 days. As shown in Figure 1*C*, active and inactive UC colonoids were delayed in establishing confluency and had lower TEER (inactive UC: UD, 700 Ω·cm^2^ ± 60; DF, 1500 Ω·cm^2^ ± 60; n = 10, *P* ≤ .05 vs HS; active UC: UD, 600 Ω·cm^2^ ± 60; DF, 1200 Ω·cm^2^ ± 80; n = 10, *P* ≤ .05 vs HS) compared with monolayers from HS (UD, 1200 Ω·cm^2^ ± 55; DF, 2500 Ω·cm^2^ ± 55; n = 10) measured at post-plating day10 for UD and day 15 for DF colonoids (Figure 1*D*). The slow growth of colonoids in 3D and 2D monolayer formation suggests that there are sustained differences within the epithelial stem cell compartment of the UC vs HS mucosa.

### Colonoids Derived From UC Tissue Form a Thin Mucus Layer and Have Defective Barrier/Mucosal Integrity

**Figure 2:**
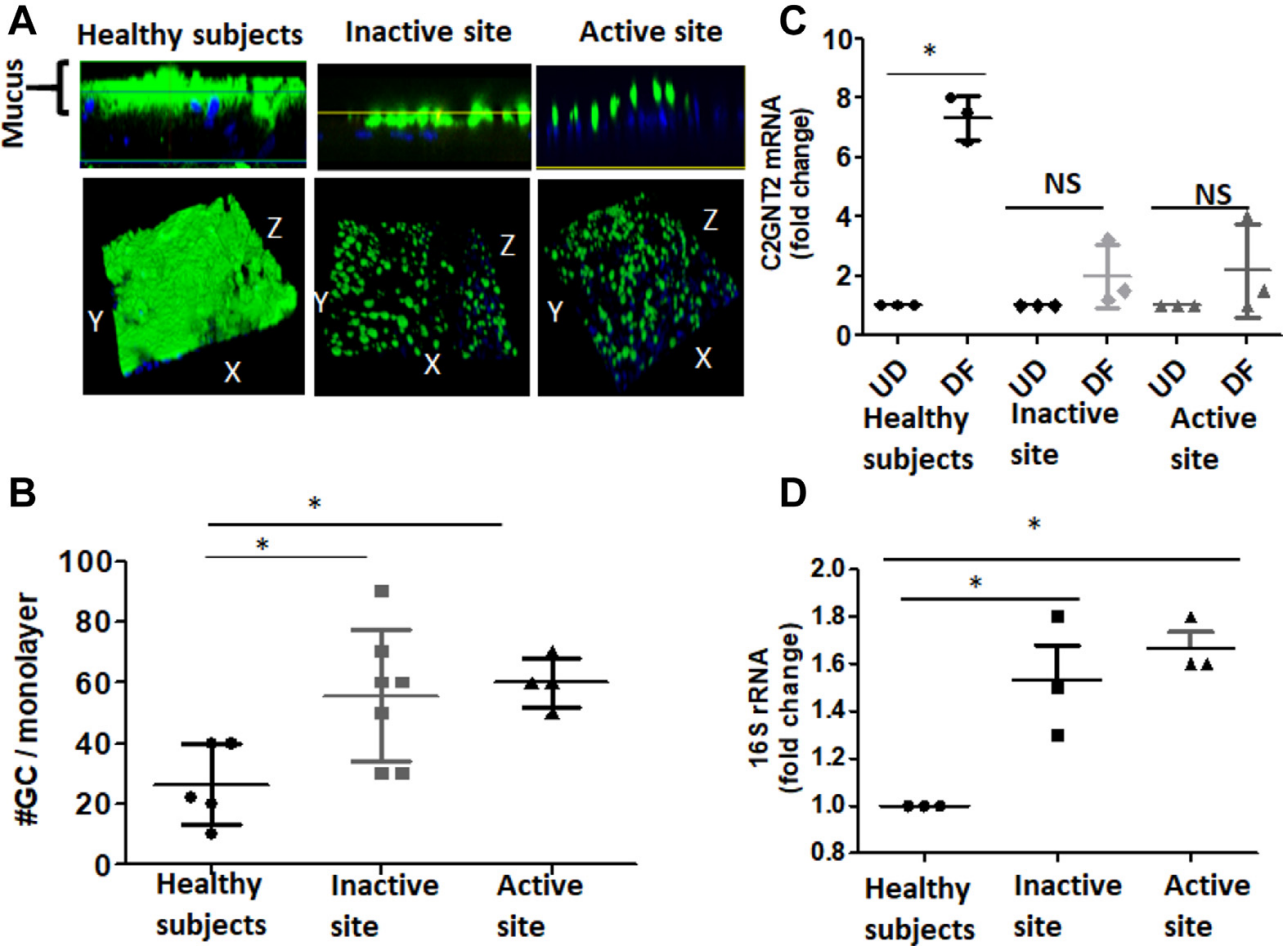
**UC colonoids have defects in mucus secretion and barrier function.***(A)* Methanol–Carnoy’s fixed DF colonoid monolayers stained with MUC2 (*green*), nucleus (*blue*). Representative confocal XZ (*above*) and 3D-XYZ (*below*) projections depicting the MUC2 layer in colonoids monolayer is shown. *(B)* Average number of GCs expressed after 5 days of differentiation of colonoid monolayers. *(C)* Differences in mRNA expression of *C2GNT2* mRNA after differentiation of monolayers from HS and inactive and active UC sites. *(D)* Bacterial *16S rRNA* expression in colonoids after 8-hour infection of DF monolayers. (*A)* and (*B)* Multiple areas of monolayers from each subject were analyzed. (*C)* and (*D)* n = 3 monolayers from each group were analyzed at different times. Results are shown as mean ± SEM. ∗*P* < .05 vs HS. Scale bar = 20 μm.

Active UC tissues have a reduced mucus layer, and many UC colons have a reduced number of mucus containing GCs.^13^ Similarly, colonoid monolayers made from the tissue derived from either inactive or active sites of UC lacked a uniform mucus layer; instead, they have a thin and non-uniform mucus layer (Figure 2*A*). We further analyzed the number of GCs in these monolayers by counting MUC2 positive cells per monolayer. Surprisingly, DF UC monolayers from both active and inactive sites had a significantly higher number of GCs compared with monolayers from HS (Figure 2*B*). The primary component of the mucus layer is MUC2, an extensively O-glycosylated molecule that forms polymeric sheets to which luminal bacteria attach and which provides a food source for the microbiota.^8^^,^^14^ O-glycans contribute to about 80% of its mass and therefore are an important determinant of mucus properties. O-glycosylation of MUC2 occurs post-translationally in the Golgi apparatus. The primary enzymes in this process are the core 1 β1,3-galactosyltransferase (C1galt1), core 2 β1, 6*N-*acetylglucosaminyltransferases (C2GnTs), and core 3 β1,3-N-acetylglucosaminyltransferase (C3GnT).^15^ The mRNA levels of several enzymes responsible for glycosylation of mucin dimers were measured including *C1galt1*, *C2GnT,* and *C3GnT*. Of the enzymes tested, *C2GNT2* did not increase with differentiation of UC colonoids as occurred in HS colonoids. Similar results were seen in colonoids from inactive and active sites of UC patients (Figure 2*C*). In contrast, mRNAs of *C1galt1* and *C3GnT* were not significantly different from HS (data not shown).

We further investigated the barrier (mucosal) integrity by exposing DF colonoid monolayers with intact mucus to apical *Escherichia coli* (1 × 10^6^ colony-forming units/mL) (8 hours) and performed 16S bacterial rRNA based real-time polymerase chain reaction (PCR) analysis on total RNA extracted from monolayers. An increased amount of bacterial 16S rRNA was present in monolayers from UC patients (inactive and active) as compared with HS, suggesting that UC colonoids have a defective mucus barrier (Figure 2*D*).

### Activation of Secretory Lineage Differentiation in UC Compared With Non–IBD Controls

**Figure 3:**
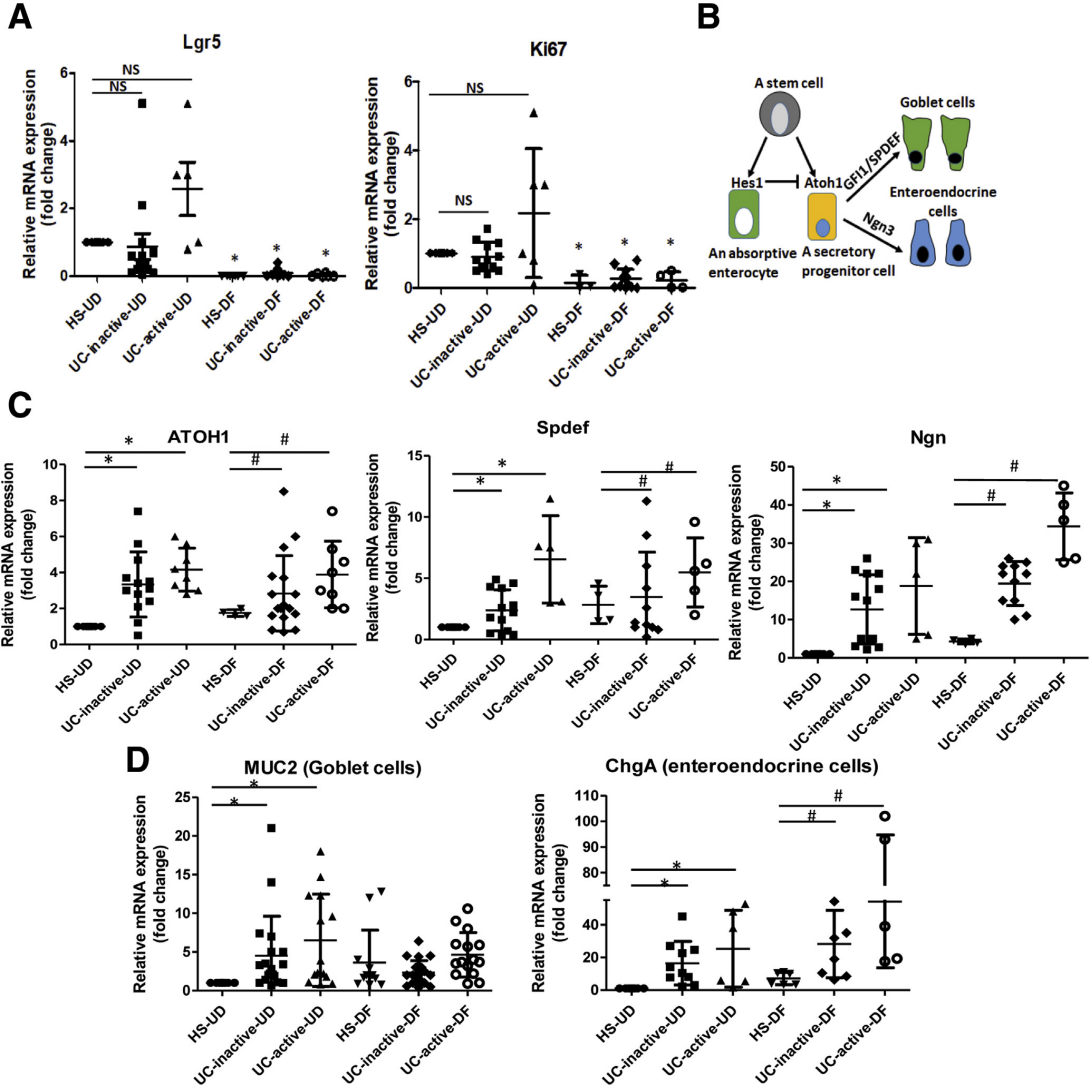
**Differential gene expression profiles in UD and DF colonoids from HS compared with UC patients: Relative mRNA levels of *(A)* proliferation genes, *(B)* schematic representation of absorptive and secretory pathways starting from a progenitor and the genes involved in this process, *(C)* secretory lineage genes, and *(D)* genes specific to different cell types, by quantitative PCR.** Messenger RNA levels are normalized to 18S ribosomal RNA expression. Result is normalized to HS set as 1 and expressed as fold change. Results are shown as mean ± SEM. ∗*P* < .05 vs HS-UD; ^#^*P* < .05 vs HS-DF; 3D colonoids from each subject were analyzed at least 2 times and used as n = 1.

To investigate the differentiation status and GC-related gene expression in UC colonoids, quantitative PCR expression analysis of a selected panel of genes was performed in UD and DF colonoids from HS and UC patients. The expressions of the stem cell gene *Lgr5* and cell proliferation marker *Ki67* were slightly but not significantly increased in both inactive and active UC colonoids compared with HS (Figure 3*A*). Nonetheless, the expression of both *Lgr5* and *Ki67* decreased with DF of UC colonoids as in HS. In addition, the expression of genes associated with mucus-producing GCs was determined. Shown in Figure 3*B* are results for a transcription factor, atonal homolog 1 (*ATOH1*), which is a gatekeeper that controls the fate of intestinal progenitors. Intestinal progenitors with reduced Notch activity express high levels of *ATOH1* and commit to a secretory lineage fate (Figure 3*B*). Therefore, *ATOH1* expression in UD and DF colonoids was measured. Both active and inactive UC colonoids in UD, as well as DF, states had significantly higher expression of *ATOH1* compared with HS (Figure 3*C*, left). The expression of transcription factors downstream of *ATOH1* were also analyzed including *SPDEF* (SAM pointed domain containing Ets transcription factor) and *Ngn3* (Neurogenin 3), which specify differentiation and maturation of GC and enteroendocrine cells, respectively (Figure 3*B*). Similar to *ATOH1*, the expressions of *SPDEF* and *Ngn3* were significantly higher in both active and inactive UC colonoids compared with HS (Figure 3*C*, middle and right, respectively). The expression of *MUC2* (GC marker) and *ChgA* (enteroendocrine cells) was also determined (Figure 3*D*). *MUC2* message was increased in the UD colonoids from both active and inactive UC compared with HS, whereas the message was not significantly different between DF colonoids from each group. In contrast, *ChgA* transcripts followed a pattern of up-regulation in UD as well as in DF UC colonoids from active and inactive UC compared with HS.

### UC Colonoids Differentially Express Ion Transport Proteins as Compared With HS

**Figure 4:**
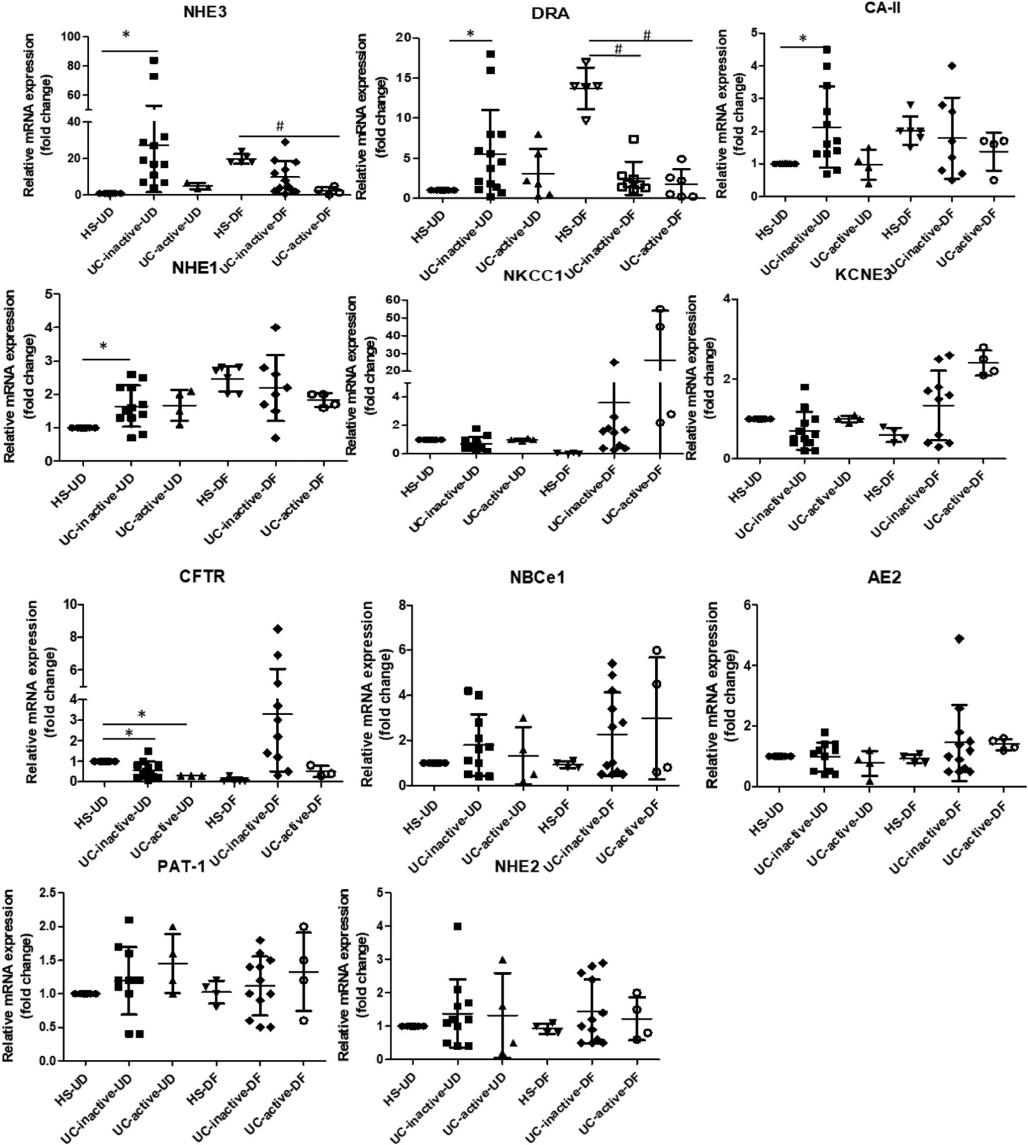
**The mRNA levels of selected ion transporters and carbonic anhydrase in UC colonoids compared with HS.** The mRNA levels of selected ion transporters were determined by quantitative real-time PCR, and relative fold changes between UD and DF colonoids were calculated using *18S ribosomal RNA* as endogenous control. Results are normalized to HS set as 1 and expressed as fold change. Spheroids from each subject were analyzed at least 3 times and used as n = 1. Means ± SEMs are shown. ∗*P* < .05 vs HS-UD; ^#^*P* < .05 vs HS-DF.

To further define the differentiation states of UC colonoids, mRNA expression of several ion transport proteins and a carbonic anhydrase isoform was determined. The ion transporters selected for study and the carbonic anhydrase isoform are known to play important roles in Cl^-^ and HCO_3_^-^ secretion, electroneutral Na^+^ absorption, and intracellular pH regulation under physiological and pathophysiological conditions and have been shown to change in expression with differentiation in intestinal epithelial cells.^16^ As shown in Figure 4, in colonoids from HS up-regulation occurred in sodium hydrogen exchanger-3 (*NHE3)* (18.4-fold), *DRA* (13.6-fold), *CA2* (2.0-fold), and *NHE1* (2.7-fold). In contrast, several ion transporters were down-regulated significantly after differentiation, including *NKCC1* (20.1-fold), potassium channel, voltage-gated, subfamily E, regulatory subunit 3 (*KCNE3*) (4.2-fold), and *CFTR* (12-fold). In contrast, UC colonoids exhibited somewhat different mRNA expression patterns compared with HS. In the UD state, UC colonoids (inactive and active site) had significantly higher expression of *NHE3* (inactive 27-fold; active 3.4-fold), *DRA* (inactive 5-fold; active 3.2-fold), and *CA2* (inactive 2.1-fold; active 1.2-fold) and lower expression of *CFTR* (inactive 0.5-fold; active 0.3-fold). Differentiation failed to cause a significant change in the expression pattern of NHE3 and DRA. Importantly, when compared with DF HS, DF UC colonoids had significantly lower expression of *NHE3* (inactive 2-fold; active 4-fold) and *DRA* (inactive 5.5-fold; active 8-fold). The mRNA levels of several other transporters were not significantly different between the groups: anion exchanger 2 (*AE2*), electroneutral Na^+^/HCO_3_^-^ co-transporter 1 (*NBCe1*), *NHE2,* and putative anion transporter 1 (*PAT-1).* Overall this suggests that in UD conditions, UC colonoids were partially differentiated based on the increased mRNA expression pattern of *NHE3*, *DRA*, and *CA-II* and decreased *CFTR* expression. In contrast, in DF colonoids from inactive and active UC, there was no further or even reduced differentiation based on reduced *NHE3* and *DRA* and a slight increase (not significant) in *NKCC1* expression. These data show that the pattern of differentiation and expression of multiple ion transporters and a carbonic anhydrase isoform in UC colonoids are different from HS.

### Defects in Mucus Layer in UC Colonoids Are Not Due to Decreased Expression of Chloride/Bicarbonate Exchanger (DRA)

**Figure 5:**
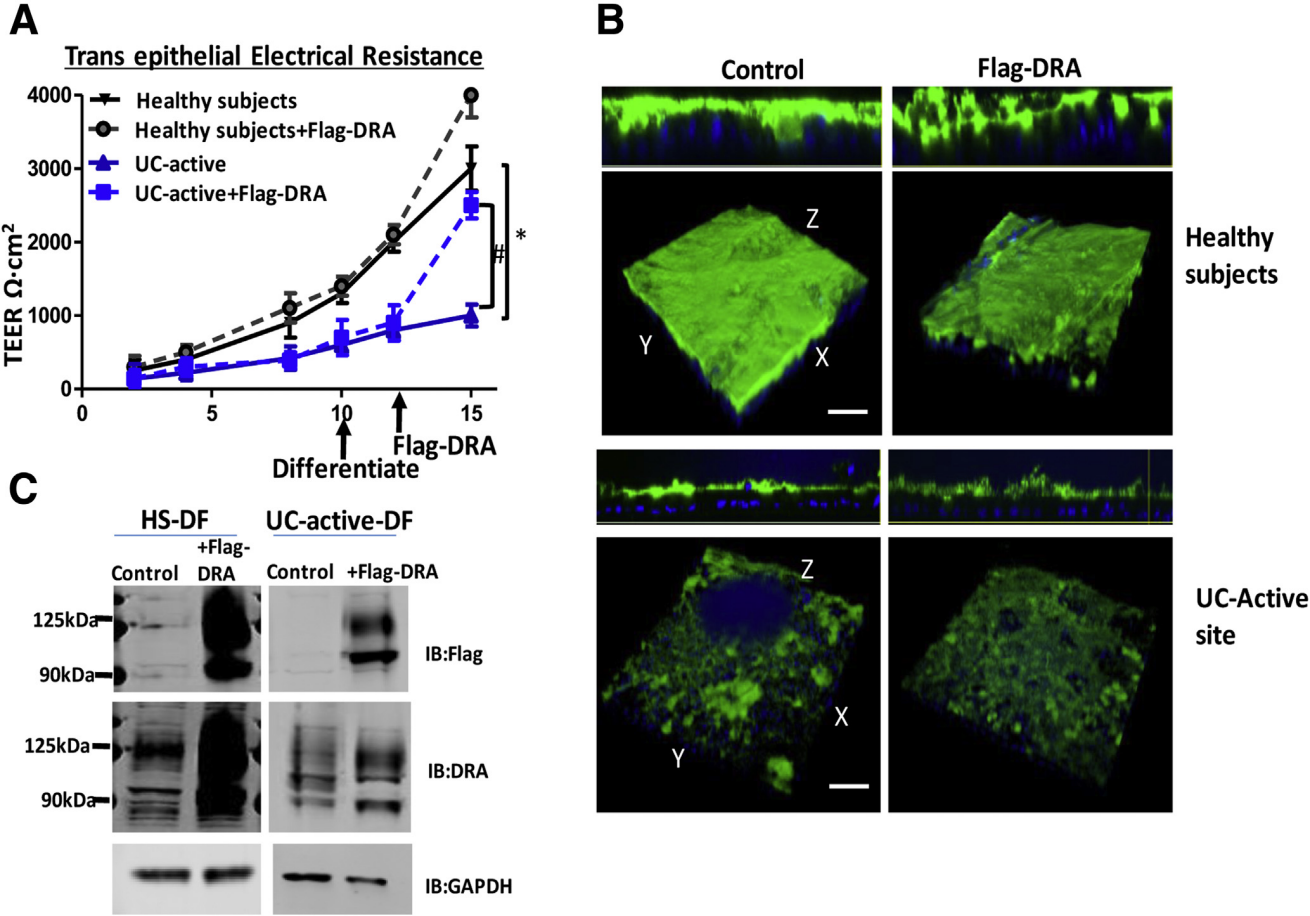
**Defects in mucus layer in UC colonoids are not due to decreased expression of DRA.***(A)* Changes in TEER of colonoid monolayers from HS (*black triangle*), and active UC (*blue triangle*) infected with adevovirus-3xflag-DRA HS (*grey circle*) and active UC (*blue square*). *(B)* Methanol–Carnoy’s fixed differentiated colonoid monolayers from HS and UC active stained with MUC2 (*green*), nucleus (*blue*). Representative confocal XZ (*above*) and 3D-XYZ (*below*) projections depicting the MUC2 layer in colonoids monolayer are shown. *(C)* Protein lysate prepared from colonoid monolayers uninfected or infected with 3x-flag-DRA was subjected to Western blot analysis and probed with DRA for endogenous expression and flag for overexpressed DRA. Representative results from 3 independent experiments with similar results are shown. Results are shown as mean ± SEM. ∗*P* < .05 vs HS; ^#^*P* < .05 vs UC control.

The presence of bicarbonate has been known to be critical for normal mucus secretion by its ability to sequester calcium from condensed mucins being discharged from GCs.^17^, ^18^, ^19^ Differentiated UC colonoids expressed GCs and failed to create a thick mucus layer, which suggested defective bicarbonate secreted into the apical domain in proximity to the mucus could be a possible etiologic feature responsible for the GC defect. Therefore, we investigated the effect of exogenously expressed chloride/bicarbonate exchanger DRA (Adeno-3xFlag-DRA) on mucus layer formation in UC colonoids. Interestingly, UC active monolayers showed a significant increase in TEER in inserts infected with Adeno-3xFlag-DRA (Figure 5*A*). Despite increasing the DRA expression to the endogenous level of DRA expression in HS, UC active monolayers failed to show any difference in the overall thickness of mucus (Figure 5*B* and *C*). These results indicate that the mucus secretion defect in UC monolayers is not due to a defect in DRA-dependent bicarbonate secretion.

### GCs in UC Colonoids Do Not Respond to Carbachol and PGE_2_ Mediated Mucin Secretion

**Figure 6:**
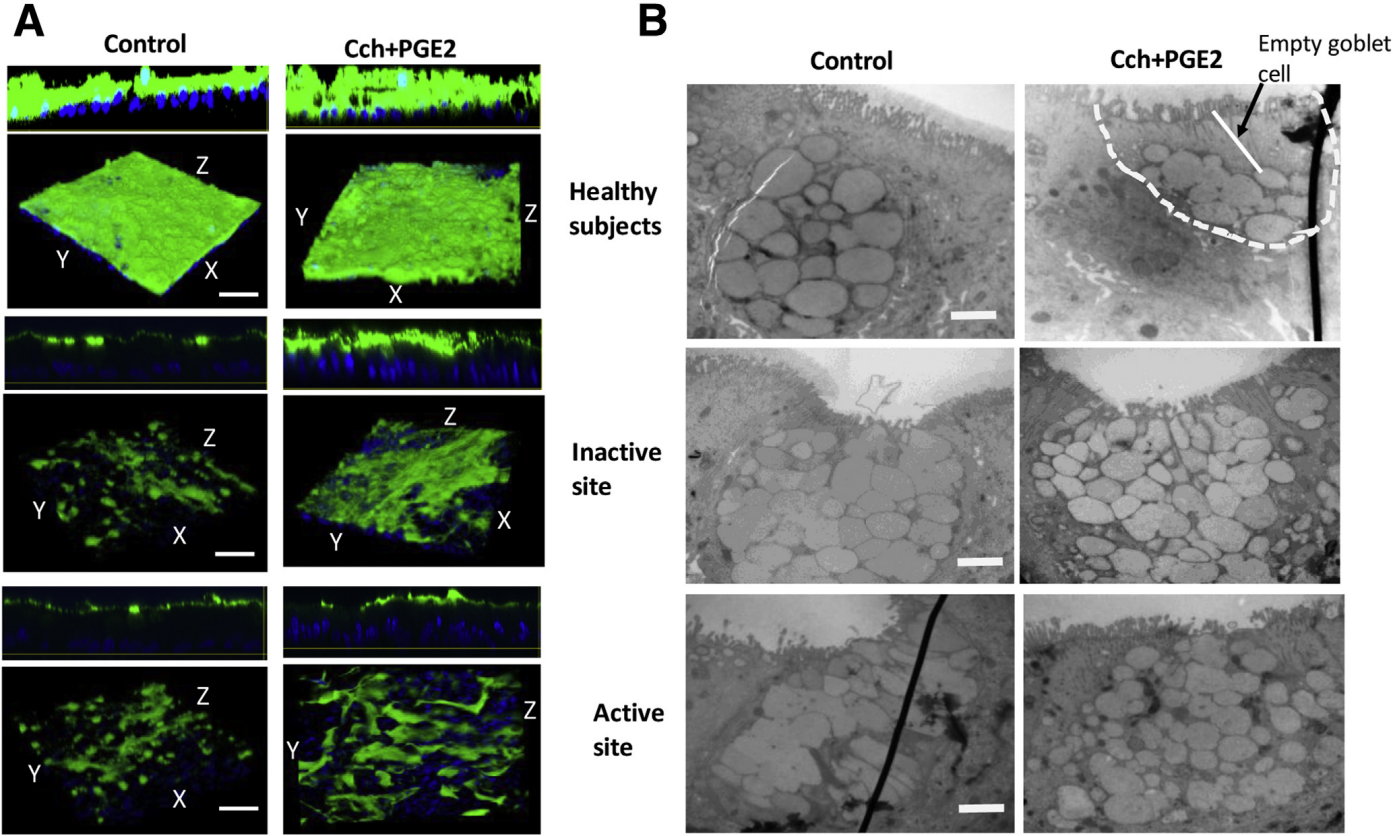
**UC colonoids have defects in mucus secretion.** Colonoid monolayers from HS and inactive and active UC sites were treated with Cch (25 μmol/L) + PGE_2_ (1 μmol/L) for 15 minutes and then analyzed. Representative image from each group is shown. *(A)* Methanol–Carnoy’s fixed monolayer stained for mucus layer, Muc2 (*green*), nucleus (*blue*). Scale bar = 20 μm. *(B)* TEM of GCs from control and Cch/PGE_2_ treated monolayers from different groups. Note empty area on apical side of GCs in HS, treated with Cch/PGE_2_, but not in UC. n = 3 monolayers from different subjects in each group. Scale bar = 500 nm.

In addition to synthesizing MUC2, GCs release stored MUC2 granules in response to cholinergic plus cyclic adenosine monophosphate (cAMP)–related stimuli. Multiple studies have found that Ca^2+^ signaling is required for the release of mucin-filled vesicles.^20^^,^^21^ Following the known muscarinic cholinergic signaling pathway for mucin secretion, we treated UC monolayers with carbachol (Cch) (25 μmol/L) to elevate intracellular Ca^2+^ and with PGE_2_ (1 μmol/L) to elevate cAMP.^22^ In contrast to Cch/PGE_2_ induced mucin secretion and creation of a thick mucus layer in colonoids from HS, monolayers from both inactive and active UC did not respond to the treatment (Figure 6*A*). At the ultrastructure (transmission electron microscopy [TEM]) level, GCs in HS had the expected appearance of granule-filled vesicles located just apical to the nucleus (Figure 6*B*). After stimulation with Cch + PGE_2_, most of the GCs in HS exhibited cavitation at the apical side. In contrast, GCs in UC monolayers (both inactive and active) did not show any decrease in the mucin vesicles in response to the treatment. Overall, these results suggest that colonoids in UC can differentiate to GCs but have a compromised secretory function in response to cholinergic/cAMP stimulation.

### TNF-α Treatment Reduces GC Number

**Figure 7:**
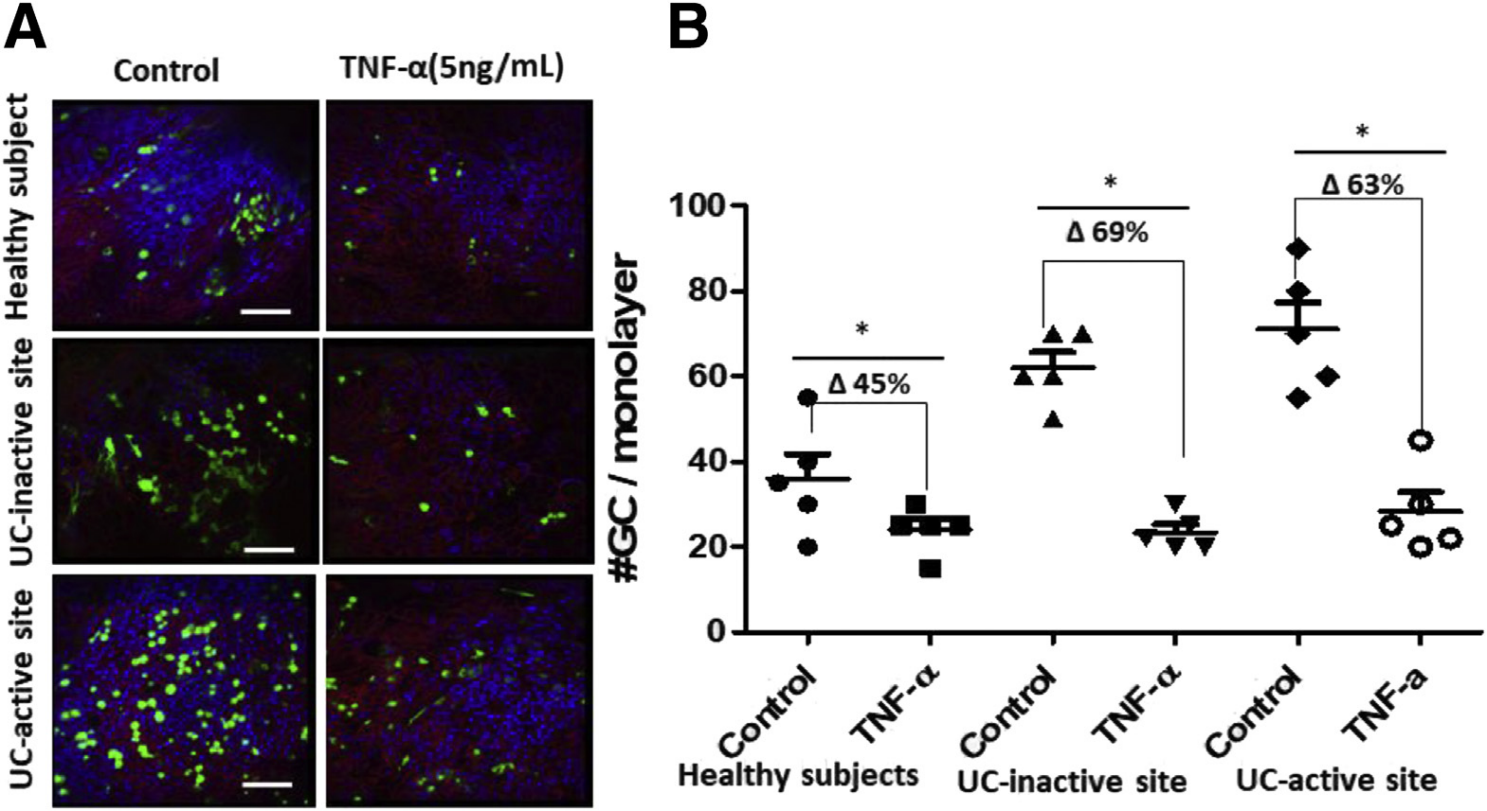
**TNF-α (5 ng/mL, 5 days) treatment decreases GC number.***(A)* Monolayers from HS and UC inactive and active sites were differentiated alone or with TNF-α (5 ng/mL) for 5 days, and MUC2 positive GCs (*green*) were analyzed using confocal imaging. TNF-α (5 ng/mL) was refreshed during medium change at second day of 5-day period. *(B)* Average number of GCs expressed in untreated or TNF-α treated monolayers. Results are shown as means ± SEMs.∗*P* < .05 vs control/untreated monolayers, n = 3 separate monolayer from each group. Scale bar = 20 μm.

Because the colonoid model is devoid of any immune cells, we hypothesized that the differences in GC number in our model from those reported in UC patient tissue samples are because of the absence of active inflammatory cytokines secreted by immune cells in UC patients. To test this hypothesis, we differentiated monolayers from HS and UC patients in the presence of an inflammatory cytokine TNF-α (5 ng/mL for 5 days, refreshed with medium change at the second day of 5-day differentiation), followed by an analysis of MUC2 positive GCs per monolayer. A representative example is shown in Figure 7*A,* and quantitation of multiple monolayers is shown in Figure 7*B*; TNF-α treated monolayers had decreased numbers of MUC2 positive GCs in both HS (control: 40 ± 12; TNF-α: 22 ± 5.6) and UC colonoids (inactive control: 65 ± 15; TNF-α: 20 ± 12; active control: 71 ± 14; TNF-α: 26 ± 10). The percent change in the number of MUC2 positive GCs in UC was higher than in HS (UC inactive, 69%; UC active, 63%; HS, 45%). These results suggest that the decrease in mucus-filled GC number reported in UC patient tissue samples is dependent on the presence of active inflammatory cytokines.

## Discussion

In this study, we provide new mechanistic insight into the basis for the reduced mucus layer that is part of the pathophysiology of UC. Although a reduced number of GCs, as reported in many UC patients, is considered as the sole cause of the reduced mucus layer, our studies suggest that the reduced mucus layer seen in UC patients is related to both reduced number and reduced secretory function of the remaining GCs. Furthermore, we also provide evidence that the epithelial compartment in UC undergoes alterations and has reduced expression of the bicarbonate transporters DRA and CFTR. Reduction in luminal HCO_3_^-^ is already known to contribute to the failure of the mucin to unfold.

Altered characteristics of epithelial cells in UC are thought to be largely due to the inflammatory environment. However, it was not known which of these changes revert to normal once the inflammation is removed, or whether some of them are imprinted in the epithelial compartment. In the present study, we took advantage of the ability to establish stem cell-derived colonoids from active and inactive areas of UC that could be passaged at least 40 times and studied them in both UD crypt-like and DF upper crypt and surface cell state to begin defining some of these long-term changes. Colonoids made from active and inactive areas of UC had properties distinct from colonoids made from the same colonic segments from healthy control subjects including the growth rate, which was slower in colonoids from active UC, and the TEER was significantly reduced in colonoids from both active and inactive UC. The reduced TEER is an indication of abnormal tight junctions and intestinal barrier function and duplicates a feature known to be present in patients with UC. Moreover, UC colonoids also showed decreased mucosal barrier integrity, which is based on an increased amount of bacterial *16S-rRNA* compared with HS. This is partially supported by a previous study showing that active UC was associated with a thin mucus layer that was penetrable with beads of a size to mimic bacteria, indicating differences in mucus quality.^23^ UD active and inactive UC colonoids had increased mRNA expression of proteins normally present in DF colonocytes including *NHE3*, *DRA*, and *CA-II* but had reduced expression of *CFTR,* which is usually more highly expressed in the crypt; moreover, when the colonoids were exposed to conditions that led to differentiation in colonoids from HS, these genes either failed to increase or decreased in expression. MUC2 also behaved similarly and in a distinctly abnormal pattern, being increased in UD active and inactive UC colonoids, whereas there was no further increase with the application of differentiation conditions. Decreased expression of DRA is reported in various inflammatory diarrhea and in UC patients.^24^ Similarly, decreased mRNA expression of *CFTR* in UC colonoids is in accordance with reports from UC patients.^25^ These results are consistent with long-term effects of inflammation in UC colonoids exhibiting early differentiation, which fits with the reduced proliferation shown in Figure 1*B*. However, the mechanisms for these long-term changes have not been identified.

The UC colonoids had an increased number of GCs compared with HS colonoids. This was consistent with the increased level of *ATOH1*, a transcription factor that increases stem cell differentiation toward the secretory pathway. Moreover, there was also an increase in *Ngn3* and *ChgA* mRNA in DF UC colonoids, another part of the ATOH1 driven secretory cell developmental pathway. Several studies have reported greater numbers of enteroendocrine cells in the colonic mucosa of patients with active UC, indicating similarity between the UC colonoid model and intact colon.^26^^,^^27^ Despite higher expression of GCs, both active and inactive UC colonoids formed a thin mucus layer, suggesting defects at the level of the signaling pathways or secretory machinery required for mucus secretion.

Secretion of mucin release from GC was examined by exposure to the muscarinic agonist Cch plus the cAMP agonist PGE_2_, which are known to cause mucin exocytosis.^28^ Formation of a functional mucus layer is a result of a complex multi-step process. It starts with an increase in intracellular Ca^2+^ in response to activation of muscarinic M3 receptors, which is followed by fusion of mucin-containing vesicles, compound exocytosis, and finally mucin unfolding via HCO_3_^-^ exposure. Mucin release was markedly reduced in both active and inactive UC colonoids based on the thickness of mucus layer in colonoid monolayers and by examining the apical area of GCs by TEM (Figure 6). This has been previously shown in ex vivo human biopsies from active UC.^29^ Further studies are required to understand which of the multiple steps in mucus secretion is abnormal in UC. The second contributor to a thin mucin layer in UC colonoids is related to the dependence of mucus unfolding on luminal HCO_3_^-^. Although it is not known whether the HCO_3_^-^ comes from adjacent epithelial cells or is more closely associated with the GCs, the mRNAs of both *DRA* and *CFTR,* the 2 major colonic apical HCO_3_^-^ transporters, were significantly reduced in DF UC colonoids. The third likely contributor to abnormal GC mucin secretion is an abnormal expression of *C2GnT2*, an enzyme responsible for O-glycosylation of MUC2. *C2GnT2* is highly expressed in the mouse small intestine and colon, and its deficiency reduces levels of core 2 and 4 O-glycans, as well as I-branching. Moreover, *C2GnT2*^*-/-*^ mice exhibit increased susceptibility to dextran sulfate sodium–induced colitis.^30^ Additional studies are required to determine whether mucin glycosylation is abnormal in UC colonoids and to define the consequence of altered glycosylation on mucus layer formation.

A thin and defective mucus layer is a signature of UC, and this has been attributed to the reduced number of colonic GCs. In contrast, in our studies, UC colonoids had an increased number of GCs compared with HS colonoids. One of the limitations of studies with stem cell-derived intestinal organoids is that they only contain epithelial cells and lack the many additional cell types present in the normal intestine, including inflammatory and immune cells. Consequently, disease models using colonoids do not entirely duplicate the inflammatory or immune environment that plays a critical role in the pathophysiology of many gastrointestinal diseases including IBD. To deal with this limitation, co-culture with additional cell types has been developed, and the use of iPSC-derived organoids includes some of the additional intestinal mesenchymal cells. Based on this limitation, we hypothesized that the difference in GC number in UC tissue compared with UC colonoids might be due to a lack of the inflammatory environment. When colonoids were exposed to TNF-α for 5 days, there was a decrease in GC number in both HS and UC colonoids, with the reduction in number in the UC colonoids exceeding that in HS. This finding supports the interpretation that the reduced number of MUC2 positive GCs in UC is at least in part due to the local inflammatory environment. The conclusion from these studies is that the reduced protective mucus layer in UC is a consequence of both a reduced number of GCs, which appears to be a reversible inflammation-dependent phenomenon, and reduced mucin secretion by the remaining GCs, which appears to be a long-term aspect of the disease.

As demonstrated in a recent study, there are multiple types of GCs in the colon that are based on their location along the crypt-surface cell axis of the mouse and human colon.^31^ In the mouse colon, the most differentiated GCs, indicated by *Ulex europaeus* agglutinin I positivity and wheat germ agglutinin negativity, are localized on the surface epithelium between the crypts called intercrypt GC. Based on their gene expression profile these GCs show a functional profile with response to stress, cell differentiation, apoptosis, and protein transport. In humans, a fewer intercrypt GC population and mucus alteration are reported in both active and in remission UC.^31^ Thus, in addition to our findings, these results suggest that long-term inflammation in UC alters the GC population such that it differs from that in HS. Further studies are required to characterize the GC population in UC colonoids.

The current observations further support several recent studies that have suggested that epithelial cells from the involved colonic mucosa of patients with UC acquire a unique transcriptional signature that is maintained long after the acute inflammation has resolved, suggesting permanent epithelial cell changes.^7^ Epigenetic changes in genes from UC mucosa have been suggested related to pathways that affect antigen processing and presentation, cell adhesion, B- and T-cell receptor signaling, JAK-STAT signaling, and transforming growth factor-beta signaling. However, the extent and consequences of epigenetic changes in IBD have not been adequately characterized. However, because abnormal barrier function and a reduced protective mucus layer contribute to the initiation of IBD and potentially to recurrence, the presence of both characteristics in colonoids over multiple passages and in colonoids made from inactive as well as active UC tissue suggests that UC mucosa is primed for recurrence even in the absence of inflammation. We speculate that an approach to reverse these changes in UC colonoids has the potential to prevent UC recurrence and potentially to prevent the proximal spread of UC, concerning and unmet needs in UC management.

## Materials and Methods

Chemicals and reagents were purchased from Thermo Fisher (Waltham, MA) or Sigma-Aldrich (St Louis, MO) unless otherwise specified. All authors have had access to the study data and reviewed and approved the final manuscript.

### Patient Population and Biopsy Collection

Colonic biopsies were obtained from HS and UC patients (Table 1) undergoing colonoscopies or from patients having colonic surgery for refractory UC. In all cases, informed consent was obtained using an experimental protocol approved by the Johns Hopkins University Institutional Review Board (IRB# NA_00038329). All procedures were performed under approved guidelines and regulations. Intestinal biopsies were collected from the ascending colon, descending colon, or sigmoid colon of HS screened with colonoscopy for colorectal cancer or gastrointestinal symptoms and who had histologically normal colon. Seven UC patients had biopsies taken from area of uninvolved mucosa and/or active disease (paired biopsies from 4 patients and from inactive sites of 3 separate UC patients). Histologic status of the biopsies from colonoscopy or surgical samples is listed in Table 1**.** UC activity at the time of the colonoscopy was categorized according to the Mayo endoscopic subscore.^32^ Active UC was defined as Mayo endoscopic subscore of ≥1; inactive disease was defined as Mayo score of 0 in a previously involved segment. Colonoids were established via the Hopkins Conte Basic and Translational Digestive Diseases Research Core Center (NIH/NIDDK P30).

**Table 1:** Clinical Descriptions and Origins of Biopsies of HS and Patients With UC

Colon lines	Known ailment	Origin of biopsies
Patient 1	UC inactive site	Descending colon
	UC active site	Rectum
Patient 2	UC inactive site	Ascending colon
	UC active site	Descending colon
Patient 3	UC inactive site	Sigmoid colon
	UC active site	Rectum
Patient 4	UC inactive site	Transverse colon
	UC active site	Sigmoid colon
Patient 5	UC inactive site	Sigmoid
Patient 6	UC inactive site	Transverse colon
Patient 7	UC inactive site	Descending colon
Healthy subject n = 2	Normal	Ascending colon
Healthy subject n = 2	Normal	Descending colon
Healthy subject n = 1	Normal	Sigmoid colon

### Organoid Culture and Monolayer Formation

Human colonoid cultures and monolayers were established by using the methods reported previously.^33^^,^^34^ Human colonoids were maintained as cysts embedded in Matrigel (Corning #356231; Corning, NY) in 24-well plates and cultured in Wnt3A, Rspon, and Noggin containing growth medium or non-differentiated medium (NDM).^34^ Medium was replaced with fresh NDM every other day. Studies were carried out on passages 6–40. For a generation of a monolayer, colonoids were fragmented in Organoid Harvesting Solution (Trevigen, Gaithersburg, MD), and multiple wells were pooled together and resuspended in NDM after centrifugation. Colonoid fragments (in 100 μL NDM) were added onto 0.4 μm pore transparent polyester membrane 24-well cell culture inserts (Transwell; Corning, or Millipore, Burlington, MA) pre-coated with human collagen IV (30 μg/mL; Sigma-Aldrich). NDM (600 μL) was added to the bottom, and the cultures were incubated at 37 °C, 5% CO_2_. Monolayers were cultured in 5% CO_2_ atmosphere at 37°C. The growth medium was supplemented with Y-27632 (10 μmol/L) and CHIR99021 (10 μmol/L) during the first 2 days after seeding. The formation of colonoid monolayers was monitored by measurement of TEER. Once monolayers became confluent, the expansion medium was replaced with a differentiation medium that was made by substituting Wnt3A, R-spondin1, and SB202190 in the expansion medium with the base medium. Five days later, paired UD and DF enteroid monolayers were studied.

### Quantitative Real-Time PCR

Total RNA was extracted from 3D cultures using the PureLink RNA Mini Kit (Life Technologies, Carlsbad, CA) according to the manufacturer’s protocol. Complementary DNA was synthesized from 1 to 2 μg of RNA using SuperScript VILO Master Mix (Life Technologies). Quantitative real-time PCR was performed using Power SYBR Green Master Mix (Life Technologies) on a QuantStudio 12K Flex real-time PCR system (Applied Biosystems, Foster City, CA). Each sample was run in triplicate, and 5 ng RNA-equivalent complementary DNA was used for each reaction. Commercially available primer pairs from OriGene Technologies (Rockville, MD) were used. The following primer pairs were used: *LGR5*: HP207145; *Ki67*: HP206104; *ATOH1*: HP208359; *NEUROG3*: HP213982; *SPDEF*: HP210328; *MUC2*: HP206138; *CHGA*: HP205193; *LYZ*: HP200222; *NHE3*: HP207529; *DRA*: HP200096; *CAII*: HP200053; *CFTR*: HP200464; *PAT1*: HP232409; *NHE1*: HP206641; *NKCC1*: HP203742; *KCNE3*: HP208601; *NBCE1*: HP232301; *AE2*: HP206636; *NHE2*: HP206642; *18S rRNA*: HP220445 (OriGene Technologies). The relative fold changes in mRNA levels of genes between DF organoids and UD organoids were determined by using the 2^-ΔΔCT^ method with human 18S ribosomal RNA simultaneously studied and used as the internal control for normalization and shown in fold change compared with the HS-UD or HS-DF control.

### Adenoviral 3xFlag-DRA Preparation, Purification, and Expression

Triple Flag-tagged human DRA was cloned into the adenoviral shuttle vector ADLOX.HTM under the control of a cytomegalovirus promoter. The virus was generated by transfection of CRE8 cells with ψ5 viral DNA and ADLOX.HTM/3Flag-DRA using Lipofectamine 2000 (Invitrogen, Waltham, MA). The crude adenoviruses were then propagated by infection in HEK 9-11 cells. Adenovirus was separated by CsCl gradient centrifugation and purified with a Sephadex G-25 column. Viral particle numbers were calculated as (*A*_260_ value) × (1.1 × 10^12^) × dilution. To test the expression of adenovirus (Adeno)-Flag-DRA, on the third day of differentiation colonoid monolayers were infected with viral particles diluted in DF medium by incubating at 37°C overnight. The next morning virus-containing medium was replaced with DF medium, and cells were allowed to grow for the next 2 days to complete 5-day differentiation.

### Immunofluorescence Staining, Confocal and TEM Image Analysis

Analysis of MUC2 by immunofluorescence and confocal microscopy was carried out as previously reported.^9^ Briefly, human colonoid monolayers were fixed with Carnoy’s solution (90% [v/v] methanol, 10% [v/v] glacial acetic acid), washed 3 times with phosphate-buffered saline, permeabilized with 0.1% saponin, and blocked with 2% bovine serum albumin + 15% fetal bovine serum for 60 minutes (all Sigma-Aldrich), followed by overnight incubation with antibodies. For immunostaining in 3D organoids, staining was done in suspension. Briefly, recovered organoids were fixed in 4% paraformaldehyde in 10 mmol/L phosphate buffer (pH 7.4) for 30 minutes at 4°C and then washed 2× with phosphate-buffered saline. Organoids were permeabilized and stained in phosphate-buffered saline with 2% bovine serum albumin, 1% Triton X-100, and 1% saponin. Images were collected using ×20 or ×40 oil immersion objective on FV3000 confocal microscope (Olympus, Tokyo, Japan) with software (Olympus) and ImageJ software (NIH). Images were 3D-reconstructed using Volocity Image Analysis software (Improvision, Coventry, England). Primary antibody rabbit anti-MUC2 (Santa Cruz Biotechnology, Dallas, TX; Cat#sc7314) was used. All antibodies were diluted at 1:100. For quantitative analysis, the same settings were used to image across samples (eg, MUC2 staining). Mucin exocytosis and thickness were determined by measuring the MUC2 positive area above the epithelial surface. For electron microscopy, 2-mm sections were fixed in 1% osmium tetroxide and 1% uranyl acetate, dehydrated with ethanol and infiltrated with epoxy resin. Thin sections (80 nm) were cut and transferred to 200-mesh copper grids before staining with uranyl acetate and lead citrate. Grids were viewed on Hitachi (Tokyo, Japna) 7600 434 TEM operating at 80 kV, and digital images of the apical regions were captured with AMT 1K × 1K CCD camera.

### Escherichia coli *Strains and Infections*

*E coli* H10407 strain was described previously.^35^^,^^36^ All antibiotics were purchased from Sigma Chemical Co (St Louis, MO). For colonoid infections, the strain was grown from frozen stocks (−80°C) at 37°C on Luria broth agar plates (Difco) 2 days before experiments. A day before infection, single colonies were inoculated in 5 mL Luria broth and grown overnight with vigorous shaking at 37°C. For infection, an overnight Luria broth culture was diluted 1:50 into fresh Luria broth and incubated at 37°C with agitation for 2 hours to achieve a log-phase culture (OD600 = 0.6). Subsequently, bacteria were adjusted to 10^8^ colony-forming units/mL in sterile phosphate-buffered saline, and 10 μL (1×10^6^) was added to the apical surface of colonoid monolayers with intact mucus. *E coli* infections were allowed to progress for 8 hours.

### Immunoblotting

Transwell inserts with or without 3xFlag-DRA infection were rinsed 3 times with phosphate-buffered saline and harvested in phosphate-buffered saline by scraping. Cell lysate preparation and Western blot were performed as previously reported.^37^ Protein bands were visualized and quantitated using an Odyssey system and Image Studio software (LI-COR Biosciences, Lincoln, NE).

### Statistical Analysis

Quantitative data are expressed as the mean ± standard error of the mean (SEM). Statistical significance was determined using analysis of variance with Bonferroni’s post-test (Prism GraphPad, San Diego, CA) to compare groups including a minimum n = 3 replicates. A *P* value less than or equal to .05 was considered statistically significant.
